# Validation of inertial measurement units based on waveform similarity assessment against a photogrammetry system for gait kinematic analysis

**DOI:** 10.3389/fbioe.2024.1449698

**Published:** 2024-08-12

**Authors:** Laura Blanco-Coloma, Lucía García-González, Isabel Sinovas-Alonso, Silvia Torio-Álvarez, Paula Martos-Hernández, Sara González-Expósito, Ángel Gil-Agudo, Diana Herrera-Valenzuela

**Affiliations:** ^1^ Biomechanics and Technical Aids Unit, National Hospital for Paraplegics, Toledo, Spain; ^2^ Biorobotics Group, CAR-Centre of Automation and Robotics, CSIC-Spanish National Research Council, Madrid, Spain; ^3^ International Doctoral School, Rey Juan Carlos University, Madrid, Spain

**Keywords:** three-dimensional (3D) kinematic gait data, inertial measurement units (IMUs), photogrammetry, waveform similarity assessment, interprotocol coefficient of multiple correlation (CMC_P_), feasibility, repeatability

## Abstract

When assessing gait analysis outcomes for clinical use, it is indispensable to use an accurate system ensuring a minimal measurement error. Inertial Measurement Units (IMUs) are a versatile motion capture system to evaluate gait kinematics during out-of-lab activities and technology-assisted rehabilitation therapies. However, IMUs are susceptible to distortions, offset and drifting. Therefore, it is important to have a validated instrumentation and recording protocol to ensure the reliability of the measurements, to differentiate therapy effects from system-induced errors. A protocol was carried out to validate the accuracy of gait kinematic assessment with IMUs based on the similarity of the waveform of concurrent signals captured by this system and by a photogrammetry reference system. A gait database of 32 healthy subjects was registered synchronously with both devices. The validation process involved two steps: 1) a preliminary similarity assessment using the Pearson correlation coefficient, and 2) a similarity assessment in terms of correlation, displacement and gain by estimating the offset between signals, the difference between the registered range of motion (∆ROM), the root mean square error (RMSE) and the interprotocol coefficient of multiple correlation (CMC_P_). Besides, the CMC_P_ was recomputed after removing the offset between signals (CMC_Poff_). The correlation was strong (r > 0.75) for both limbs for hip flexion/extension, hip adduction/abduction, knee flexion/extension and ankle dorsal/plantar flexion. These joint movements were studied in the second part of the analysis. The ∆ROM values obtained were smaller than 6°, being negligible relative to the minimally clinically important difference (MCID) estimated for unaffected limbs, and the RMSE values were under 10°. The offset for hips and ankles in the sagittal plane reached -9° and -8°, respectively, whereas hips adduction/abduction and knees flexion/extension were around 1°. According to the CMC_P_, the kinematic pattern of hip flexion/extension (CMC_P_ > 0.90) and adduction/abduction (CMC_P_ > 0.75), knee flexion/extension (CMC_P_ > 0.95) and ankle dorsi/plantar flexion (CMC_P_ > 0.90) were equivalent when captured by each system synchronously. However, after offset correction, only hip flexion/extension (CMC_Poff_ = 1), hip adduction/abduction (CMC_Poff_ > 0.85) and knee flexion/extension (CMC_Poff_ > 0.95) satisfied the conditions to be considered similar.

## 1 Introduction

Biomechanics is considered an important tool to assess gait during rehabilitation therapies, since it allows the quantitative analysis of human walking, gait features recognition, and its use for diagnostic purposes and treatment planning (Growney et al., 1997; Yavuzer et al., 2008; Xu et al., 2024). However, in technology-assisted rehabilitation therapies, evaluating the immediate biomechanical effects of using technology becomes a challenge. Multiple devices allow assessing biomechanics such as optoelectronic systems based on 3D photogrammetry and Inertial Measurement Units (IMUs) to evaluate kinematics, or force platforms in the case of kinetic analysis. Currently, in neurorehabilitation, the gold standard to assess therapy and intervention outcomes are photogrammetry systems, either with active or passive markers, as they allow in-depth analysis of gait kinematics due to their high accuracy (Eichelberger et al., 2016). Nevertheless, these systems have some drawbacks related to the quality of the recordings, which may be influenced by the number of cameras, the occlusion of markers, the time spent in the execution of the test due to instrumentation, or the expertise of the evaluator (Cano de la Cuerda and Collado Vázquez, 2012; Hassani et al., 2022). In accordance with these limitations, photogrammetry systems are not an option when performing tests to evaluate kinematics in out-of-lab environments, to study the immediate effect of rehabilitation technologies, or when therapies involve multiple devices. Therefore, as a more versatile alternative, IMUs motion capture (mocap) systems are used to evaluate gait kinematics.

IMUs are small and light motion sensor devices based on micro-electro-mechanical technology that estimate the orientation of a body segment to which they are attached from the inertial forces experienced by that segment. The orientation of the IMU is expressed with respect to a fixed coordinate system based on the magnetic north and Earth’s gravitational force. In this way, no specially equipped laboratories are necessary to use them. This makes the system portable and useable both outdoors and during technology-assisted therapies (Ferrari et al., 2010b; Francisco and Tejada, 2020; Hassani et al., 2022).

However, registration will be valid as long as the magnetic field is not distorted (de Vries et al., 2009). Besides, IMUs also suffer from drifting biases, a type of cumulative noise in their measurements that hinders an integration-based analysis to estimate kinematics (Sabatini, Ligorio, and Mannini, 2015; Dorschky et al., 2019). In recent years, multiple approaches based on filtering and global optimization have been proposed to cope with sensor noise and drift to correctly estimate the relative position and orientation of each body segment. For example, incorporating an extended Kalman filter in the sensory fusion process to obtain the corrected orientation of each sensor and segment, or applying a Gaussian distribution to model accelerometer noise and gyroscope bias (Roetenberg et al., 2005; Kok, Hol, and Schön, 2014). Nevertheless, their implementation does not always achieve drift-free estimation of joint angles. In addition, unlike photogrammetry systems, it does not consider the anthropometric measures of the users, there is no standardized placement zone, and its angular measurements always start from zero regardless of the initial posture. These lead to greater sources of error, causing offset and not recording the real joint range of motion (ROM) in their registrations, therefore biomechanical constraints are included in previous studies (Kok, Hol, and Schön, 2014; Dorschky et al., 2019). All these factors make the system susceptible to distortions, so it is important to design and validate an instrumentation and recording protocol able to control these aspects.

Reliability of gait parameters with minimal measurement error is an important consideration in the clinical use of quantitative gait analysis outcomes (Yavuzer et al., 2008), therefore gait analysis requires an accurate, reproducible and precise measurement system (Hassani et al., 2022). It is important to investigate whether a variation between measurements is a therapy effect or is solely due to variation in registrations (Hammer and Lindmark, 2003). Significant information will be lost if recording errors mask gait impairments. Therefore, before using IMUs to measure gait kinematics, it is indispensable to validate the technology with a reliable reference system. Common approaches are to use an optoelectronic mocap system as a reference due to its proven precision (Ferrari et al., 2010b; Kim and Nussbaum, 2013; Zhang et al., 2013; Schiefer et al., 2014).

For all these reasons, the aim of this study was to present a validation protocol to evaluate the accuracy of gait kinematic assessment with IMUs based on the similarity of the waveform of the signals captured by this system and those captured by a photogrammetry system synchronously during gait tests. For this purpose, concurrent measurements were taken with IMUs and the reference photogrammetry system in individuals without gait disorders, since they have a repeatable gait pattern that allows comparing the equivalence of the waveforms taken by both devices and determining the reliability of the IMUs system (Kadaba et al., 1989).

## 2 Materials and methods

### 2.1 Participants

A gait database of 32 healthy adult subjects was gathered, volunteers were between 20 and 63 y. o. (33.64 ± 12.44) and 71.88% were females. The detailed demographic and clinical characteristics of the sample are presented in Table 1. The data were collected between June and November 2023. Every individual underwent a barefoot walking test recorded simultaneously with IMUs and the photogrammetry system. The dataset contains the kinematic gait information of the hip, knee, and ankle joints in the three planes of motion: sagittal, frontal, and transversal (Blanco-Coloma et al., 2024).

**TABLE 1:** Demographic and anthropometric characteristics of the 32 subjects that make up the healthy gait database for validation.

Group N = 32		
Age, mean ± sd		33.65 ± 12.44
Gender (women/men)		23/9
Wieght (kg), mean ± sd		70.88 ± 19.91
Height (m), mean ± sd		1.69 ± 0.091
Lower limb length (mm), mean ± sd	R	883.88 ± 50.74
	L	884.97 ± 51.14
Knee width (mm), mean ± sd	R	117.56 ± 14.33
	L	117.5 ± 13.90
Ankle width (mm), mean ± sd	R	67.84 ± 5.33
	L	66.75 ± 4.63
InterASIS (mm), mean ± sd		260,5 ± 38.47
Shoulder offset (mm), mean ± sd	R	41.42 ± 5.55
	L	41.77 ± 5.56

sd, standard deviation; R, right limb; L, left limb; InterASIS, distance between anterosuperior iliac spines.

All subjects were informed of the purpose of the study, the possibility of withdrawing from the same, and signed an informed consent for gait analysis. The study protocol was approved by the local bioethics committee (Clinical Research Ethics Committee at University Hospital Complex of Toledo, CEIC-CHTO-NO 1006 of 26 April of 2023 and NO 949 of 25 January of 2023).

### 2.2 Experimental procedure and data acquisition

**FIGURE 1:**
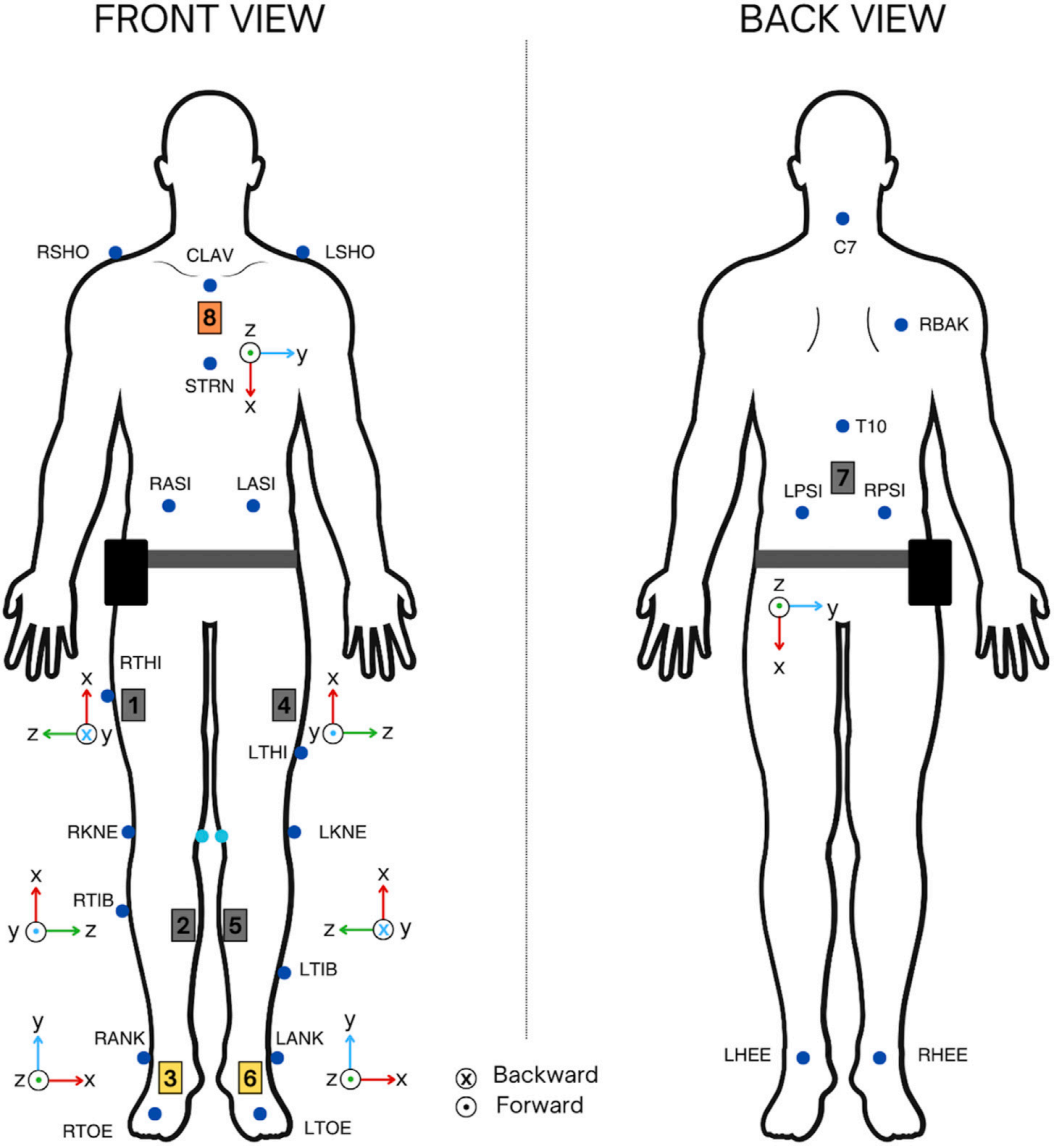
IMUs and the photogrammetry system Vicon marker set instrumentation for synchronous captures recording.

Each subject was instrumented with 8 IMUs of the Tech-MCS V3 mocap system (Technaid S.L., Spain), and with 23 passive markers of Vicon photogrammetry system (Vicon Motion System, Oxford, United Kingdom), following the Plug-in Gait marker set model (Motion Capture System, 2017; Plug-in Gait Reference Guide, 2020). Two additional markers were placed on the medial condyles to adjust femur rotation during processing (Figure 1). For each subject a maximum of 15 captures were registered.

For IMUs instrumentation, a standardized model was developed according to the anthropometric measures of each subject. Thigh sensors were placed in the upper third of the segment, displaced frontally 5 cm from the vertical line formed by the trochanter and the lateral condyle of the knee. Tibia sensors were placed in the upper third of the segment slightly displaced towards the inner part, laterally touching the tibial spine. Ankle sensors were placed in the instep, the lumbar sensor at L4, and the chest sensor on the sternum, close to the clavicle. The axes orientation of the sensors was also defined and equal between subjects. The instrumentation with IMUs and the photogrammetry marker set is shown in Figure 1.

The calibration position of the system was fixed to avoid introducing offsets in the captures. This position was as follows: arms extended in a T-shape, trunk and legs extended and opened to the width of the hips, keeping the ankles completely aligned with the tibia segment in a neutral 0° position.

### 2.3 Data analysis

#### 2.3.1 Signal processing

Gait kinematics recorded with the photogrammetry system were preprocessed with Nexus 2.10.3 software (Vicon Motion Systems, Oxford, United Kingdom). Standard processing operations combined with the anthropometric data entered in the system allowed estimating the position of the joint centers and, subsequently, obtaining the kinematic trajectories of each joint angle of the lower extremities in the three planes of motion, resulting in a personalized and accurate gait analysis. Likewise, captures recorded with the IMUs were transformed from quaternions to Euler angles with the Tech MCS software (Technaid S.L., Spain). Afterward, the trials registered with each system were exported and further processed in MATLAB_R2021b software (The MathWorks, Inc., Natick, Massachusetts, United States). The IMUs signals were smoothed with the Saviztky-Golay filter of order three and with frame length of 21 samples. The photogrammetry signals were captured with a sampling frequency of 100 Hz; therefore, these were resampled to the IMUs sampling frequency, 50 Hz, and aligned with the corresponding IMUs signal. Henceforth, in this article, the signals recorded by the IMUs are referred to as I(t) and those recorded by Vicon V(t). Then, gait cycles were extracted for each pair of trials, obtaining a pair of waveforms for each gait cycle [I(t), V(t)]. A total of 268 synchronous gait cycles [I(t), V(t)] were recovered and analyzed in the three planes of motion per limb.

#### 2.3.2 Waveform similarity assessment

To validate the robustness and feasibility of the IMUs configuration, the similarity and variability of the waveforms of the extracted gait cycles was studied.

To determine whether these two mocap devices were interchangeable (i.e., equivalent) for measuring kinematics, the evaluation of similar waveforms was performed. To assess the similarity between I(t) and V(t) in terms of correlation, displacement, and gain, four parameters were calculated for each [I(t), V(t)] and each joint-angle: their Pearson correlation coefficient (r), the offset between I(t) and V(t), the difference between the registered range of motion (∆ROM) and the root mean square error (RMSE) (Ferrari et al., 2010b). The formulas used to calculate each are presented in Equations 1–3.

$$offset=mean\left[I\left(t\right)\right]-mean\left[V\left(t\right)\right]$$ (1)

$$r=\frac{n\left(\sum xy\right)-\left(\sum x\right)\left(\sum y\right)}{\sqrt{\left[n\sum x^{2}-{\left(\sum x\right)}^{2}\right]}\left[n\sum \right.y^{2}-{\left(\sum y\right)}^{2}]\}}$$ (2)

$$∆ROM=ROM\left[I\left(t\right)\right]-ROM\left[V\left(t\right)\right]$$ (3)

In addition, the adjusted variation of the within-day coefficient of multiple correlation (CMC) of Kadaba, named as the interprotocol CMC (CMC_P_), was calculated. This parameter assesses the repeatability of kinematics removing all other sources of “gait-cycle-to-gait-cycle” variability: 1) the biological variability of the subject’s lower limb kinematics, 2) the variability in the spread of soft tissue artefact in the lower limb kinematics and 3) the variability in the performance of the measurement system (Mcginley et al., 2009; Ferrari et al., 2010a; Ferrari et al., 2010b). Given that each I(t) can be compared only with its synchronous V(t), the aim of this new CMC_P_ statistic formulation is to assess the similarity of waveform (joint angles) acquired synchronously with different protocols and different measurement systems, within each gait cycle, when the effect of the media on waveform similarity is the only parameter of interest. It considers the magnitude of the waveform data and provides a value between 0 and 1, with a value of 1 indicating perfect similarity between two waveforms, [I(t), V(t)]. The formulation shown in Equation 4 was used to evaluate the interprotocol similarity, the CMC_P._ Suppose that for a subject and a joint angle, the kinematics are measured synchronously through *P* protocols, in *G* gait cycles. Consequently, *P* waveforms are available for each *g*th gait cycle, one per protocol, each of *F*_*g*_ frames (Ferrari et al., 2010a).

$$CMC_{p}=\sqrt{1-\frac{\sum_{g=1}^{G}\left[\sum_{p=1}^{P}\sum_{f=1}^{F}{\left(Y_{gpf}-{\bar{Y}}_{gf}\right)}^{2}/GF_{g}\left(P-1\right)\right]}{\sum_{g=1}^{G}\left[\sum_{p=1}^{P}\sum_{f=1}^{F}{\left(Y_{gpf}-{\bar{Y}}_{g}\right)}^{2}/G\left(PF_{g}-1\right)\right]}}$$ (4)

Where $Y_{gpf}$ is the ordinate at frame *f* of the waveform provided by protocol *p* at gait cycle *g*, ${\bar{Y}}_{gf}$ is the ordinate at frame *f* of the average waveform among the *P* waveforms for the gait cycle *g,* and ${\bar{Y}}_{g}$ is the grand mean for the gait cycle *g* among its *P* waveforms (Ferrari et al., 2010a).

If within each gait cycle, the variability of the *P* waveforms around their mean waveform is less than the variance around their overall mean, the CMC_P_ approaches one. Otherwise, the CMC_P_ tends to zero or even turns into a complex number. This happens, for instance, when the ROM of the *P* waveforms is comparable to the phase difference (offset) among them (Kadaba et al., 1989; Ferrari et al., 2010a). To interpret the CMC_P_ and r values obtained, the following ranges were considered: poor (0–0.65), moderate (0.65–0.75), good (0.75–0.8), very good (0.85–0.95) and excellent (0.95–1) (Yavuzer et al., 2008; Ferrari et al., 2010b).

The CMC_P_ considers the overall effect of the offset, r, and gain between waveforms, but it has limitations when recording gait curves with low ROM, resulting in complex values, whose interpretation is not agreed upon, nor evident in the formula breaking down (Røislien et al., 2012). Unlike other studies that drew conclusions from validation by focusing mainly on this parameter, this protocol established a stage-by-stage analysis to identify the joint angles that have equivalent waveforms with both systems by analyzing each parameter independently and assessing how they influence the CMC_P_ value.

The first part of the waveform similarity assessment between the signals I(t) and V(t) was based on the calculation of their correlation. The analysis continued for those joint angles that meet the following requirement:

- Condition 1: The median value of r should follow a strong positive tendency (>0.7) (Kotu and Deshpande, 2019). For this reason, it was established that in the sagittal plane, the median value of r should belong at least to the very good range (0.85–0.95). In the frontal and transversal plane, the median value of r should belong at least to a good range (0.75–0.85).

Next, for those joint angles that met condition 1, the remaining parameters related to displacement and gain were calculated: the offset, the ∆ROM and the RMSE; as well as the CMC_P_. The signals I(t) and V(t) were totally equivalent, and therefore the recording systems completely substitutable, for those movement planes that satisfied the following condition:

- Condition 2: In the sagittal plane, the median values of CMC_P_ should belong to the excellent range (0.95–1). In the frontal and transversal planes, the CMC_P_ median values should belong at least to the very good range (0.85–0.95).

Conditions had higher acceptance thresholds for the sagittal plane because there is evidence that it is the plane with most reliable and repeatable kinematics, especially for the hip and knee (Kadaba et al., 1989; Mcginley et al., 2009). Furthermore, photogrammetry, the gold standard for kinematic assessment, has higher inter-trial, intra- and inter-evaluator precision and reliability in the sagittal plane than in the other two (Fonseca et al., 2023). Thus, the acceptance thresholds were lower for frontal and transversal motion planes.

The values of all parameters are presented with box-and-whisker plots as well as in terms of median and whisker range for each limb and each joint angle, allowing the variability and dispersion of the recorded data to be studied. Median and whiskers were used because all the parameters did not follow a normal distribution for all joint angles; normality was tested with Lilliefors.

Besides, for those planes that satisfied condition 2, the CMC_P_ was recomputed after zeroing the offset (CMC_Poff_) for each couple [I(t), V(t)] to measure the effect of the displacement on the similarity. The offset was corrected by subtracting from each gait cycle I(t) the offset between it and its partner V(t), that was previously calculated with Equation 1 (Kadaba et al., 1989).

At last, a visualization of the kinematics recorded is given for every joint angle before correcting the offset, including those that did not satisfy condition 1, and after removing the offset, for those that were analyzed in condition 2.

## 3 Results

### 3.1 Waveform similarity assessment: condition 1

**FIGURE 2:**
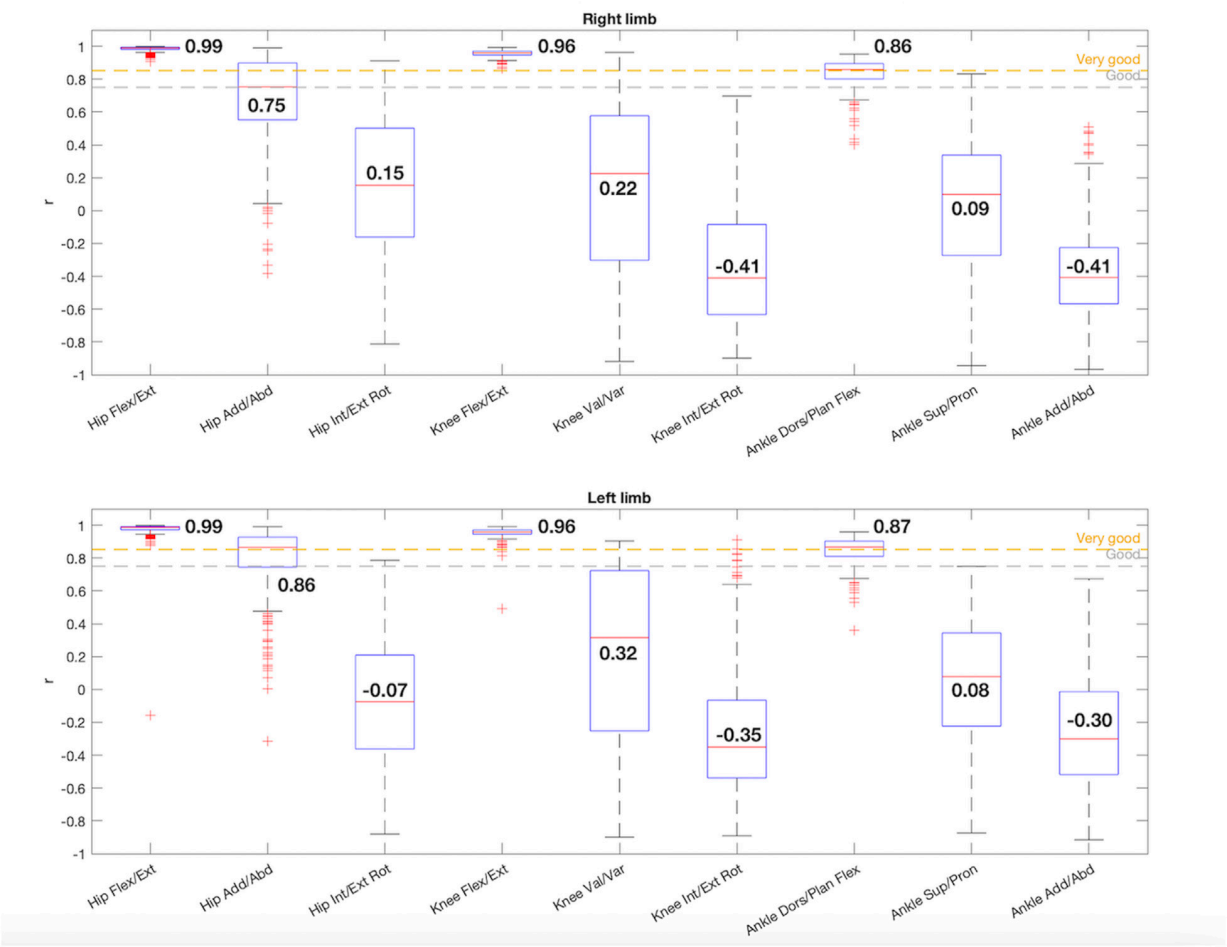
Box-and-whisker plot for r regarding the comparison between the 9 joint angles acquired with IMUs and the photogrammetry system. The median value for each joint angle is reported.

The results of the r parameter calculated for each pair of gait cycles [I(t), V(t)] are shown in Figure 2. It shows the distribution of r for each joint angle considering all acquired gait cycles. The figure shows two boxplots, one for each leg, with data from 268 pairs of gait cycles [I(t), V(t)] each. In total, each boxplot contains 268*9 = 2412 values (9 joint angles). Data are displayed in boxplots because not all parameters follow a normal distribution. The results of the normality assessment performed for each parameter with the Lilliefors test are given in Table 2.

**TABLE 2:** Results of the Lilliefors normality test computed for the parameters estimated for each pair of gait cycles [I(t), V(t)] and each joint angle.

	r p-value		ΔROM p-value		offset p-value		RMSE p-value		CMC_P_ p-value		CMC_Poff_ p-value	
	R	L	R	L	R	L	R	L	R	L	R	L
Hip Flex/Ext	.001	.001	.001	.077	.001	.001	.500	.036	.001	.001	.001	.001
Hip Add/Abd	.001	.001	.032	.011	.143	.239	.003	.001	.001	.001	.001	.001
Hip Int/Ext Rot	.008	.059	.001	.058	.133	.001	.001	.001	.001	.001	.001	.001
Knee Flex/Ext	.001	.001	.500	.001	.451	.263	.001	.001	.001	.001	.015	.001
Knee Val/Var	.001	.001	.019	.019	.286	.006	.001	.001	.001	.001	.001	.001
Knee Int/Ext Rot	.001	.001	.002	.001	.015	.004	.003	.001	.001	.001	.001	.001
Ankle Dors/Plant Flex	.001	.001	.099	.500	.001	.001	.319	.001	.001	.001	.001	.001
Ankle Sup/Prn	.003	.003	.001	.010	.010	.266	.001	.001	.001	.001	.001	.001
Ankle Add/Abd	.001	.006	.032	.500	.014	.263	.001	.001	.001	.001	.001	.001

r, Pearson correlation coefficient; ΔROM, difference in the range of motion; RMSE, root mean square error, CMC_P_, interprotocol coefficient of multiple correlation; CMC_Poff_, interprotocol coefficient of multiple correlation without offset; R, right limb; L, left limb; Flex/Ext, flexion/extension; Add/Abd, adduction/abduction, Int/Ext Rot, interior/exterior rotation; Val/Var, valgus/varus; Dorsi/Plant Flex, dorsi/plantar flexion.

To conclude that the samples are from a normally distributed population the p-value obtained must be above the 5% significance level. Not all p-values are greater than 0.05, so not all parameters are normally distributed.

For the sagittal plane, both hips had a median r value of 0.99 and both knees 0.96, showing an excellent correlation. Thus, there was a positive direct relationship between the gait cycles of each couple [I(t), V(t)], showing a similar waveform (Kotu and Deshpande, 2019). For the ankle dorsi/plantar flexion the median r values decreased slightly but were also within the very good correlation range, being 0.86 for the right limb and 0.87 for the left one. The three joints of both limbs complied condition 1.

In the frontal plane both hips had a strong correlation, showing a good correlation for the right hip adduction/abduction movement (r = 0.75) and a very good correlation for the left one (r = 0.86), satisfying condition 1, despite having more scattered data and more outliers. However, in the frontal plane for the knees (right: r = 0.22, left: r = 0.32) and ankles (right: r = 0.09, left: r = 0.08) the r medians presented low values with a high dispersion.

Finally, the hip rotation in both limbs presented a poor correlation, being the median values of r 0.15 and −0.07 for the right and left limbs, respectively. Equally, the knees (right: r = −0.41, left: r = −0.35) and the ankles (right: r = −0.41, left: r = −0.30) in the transversal plane showed no similarity between [I(t), V(t)] gait cycles and a lot of dispersion in the data.

### 3.2 Waveform similarity assessment: condition 2

The results obtained for the ∆ROM, offset, RMSE and CMC_P_ estimations are shown for those joint angles that satisfied condition 1: hip flexion/extension, hip adduction/abduction, knee flexion/extension and ankle dorsal/plantar flexion.

**FIGURE 3:**
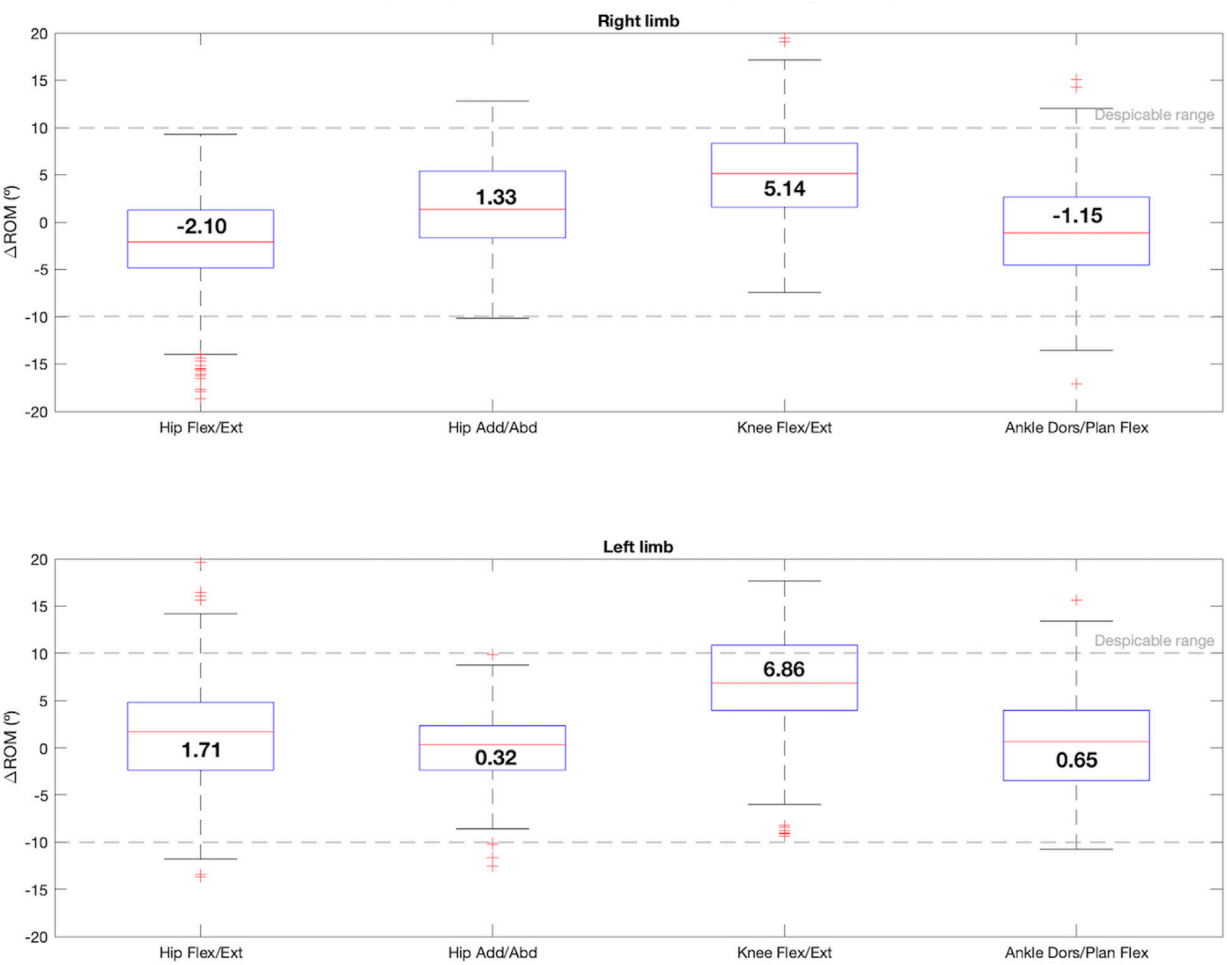
Box-and-whisker plot for ∆ROM regarding the comparison between the 4 joint angles that satisfy condition 1: hip flexion/extension, hip adduction/abduction, knee flexion/extension and ankle dorsi/plantar flexion. The median value for each joint angle is reported.

When the resulting value of the ∆ROM is negative, it means that the range measured by the IMUs was smaller than that measured by the photogrammetry system, while if it is positive, the opposite was true. As seen in Figure 3, in the sagittal plane, the median ∆ROM values for the hips were −2.10° in the right limb and 1.71° in the left one, showing slightly more dispersion and more outliers. In the case of the knees, the ROM registered by the IMUs was higher than the ROM registered by the photogrammetry for both limbs (right: ∆ROM = 5.14°, left: ∆ROM = 6.86°). For the ankles in the sagittal plane (right: ∆ROM = -1.14°, left: ∆ROM = 0.65°) and the hip in the frontal plane (right: ∆ROM = 1.33°, left: ∆ROM = 0.32°) smaller differences were observed.

**FIGURE 4:**
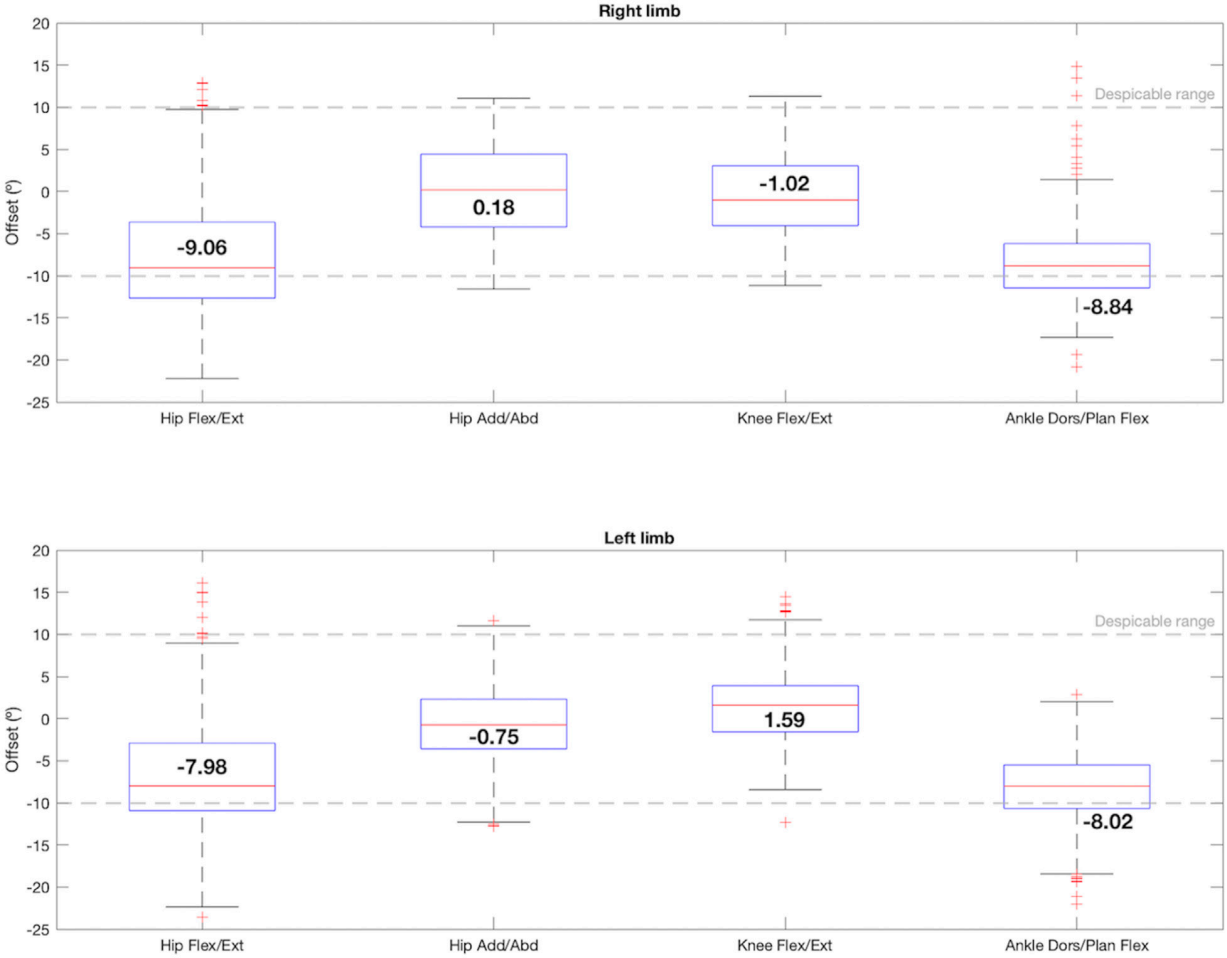
Box-and-whisker plot for offset regarding the comparison between the 4 joint angles that satisfy condition 1: hip flexion/extension, hip adduction/abduction, knee flexion/extension and ankle dorsi/plantar flexion. The median value for each joint angle is reported.

The offset box-plot graph is shown in Figure 4. According to the offset formula, when the resulting value is negative, it means that the IMUs measurement had a negative offset (lower values) with respect to the photogrammetry system, and if it is positive, the opposite happened. For the hip, the sagittal plane had a median offset value of −9.06° for the right limb and −7.98° for the left one. However, the offset in the frontal plane was lower (right: 0.18°, left: −0.75°). The knee flexion/extension also presented a small offset for both limbs, being the median values −1.02° and 1.59° for right and left, respectively. Besides, both ankles showed a negative offset of -8°. In this case, the hips and ankles in the sagittal plane had more data dispersion and outliers.

**FIGURE 5:**
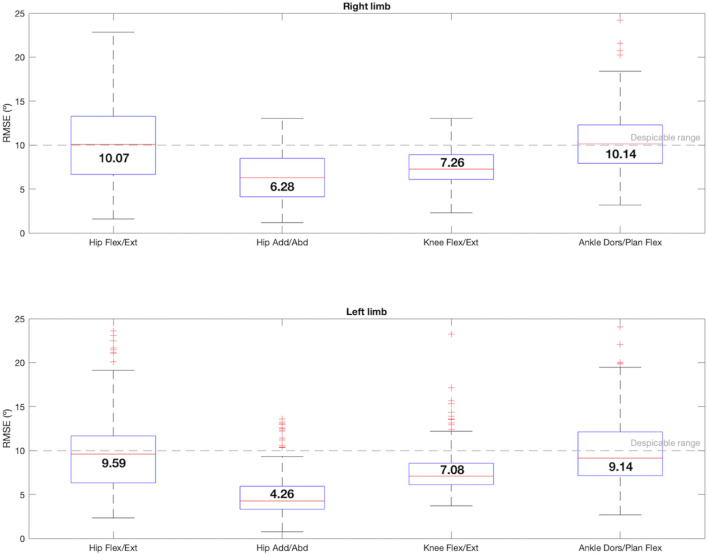
Box-and-whisker plot for RMSE regarding the comparison between the 4 joint angles that satisfy condition 1: hip flexion/extension, hip adduction/abduction, knee flexion/extension and ankle dorsi/plantar flexion. The median value for each joint angle is reported.

Analyzing the RMSE, depicted in the Figure 5, both hips and ankles in the sagittal plane presented more scattered data and the median RMSE values were around 10°. The knees presented a median error of 7°, and the hip adduction/abduction movement showed an error of 6.28° in the right leg and 4.26° in the left leg.

**FIGURE 6:**
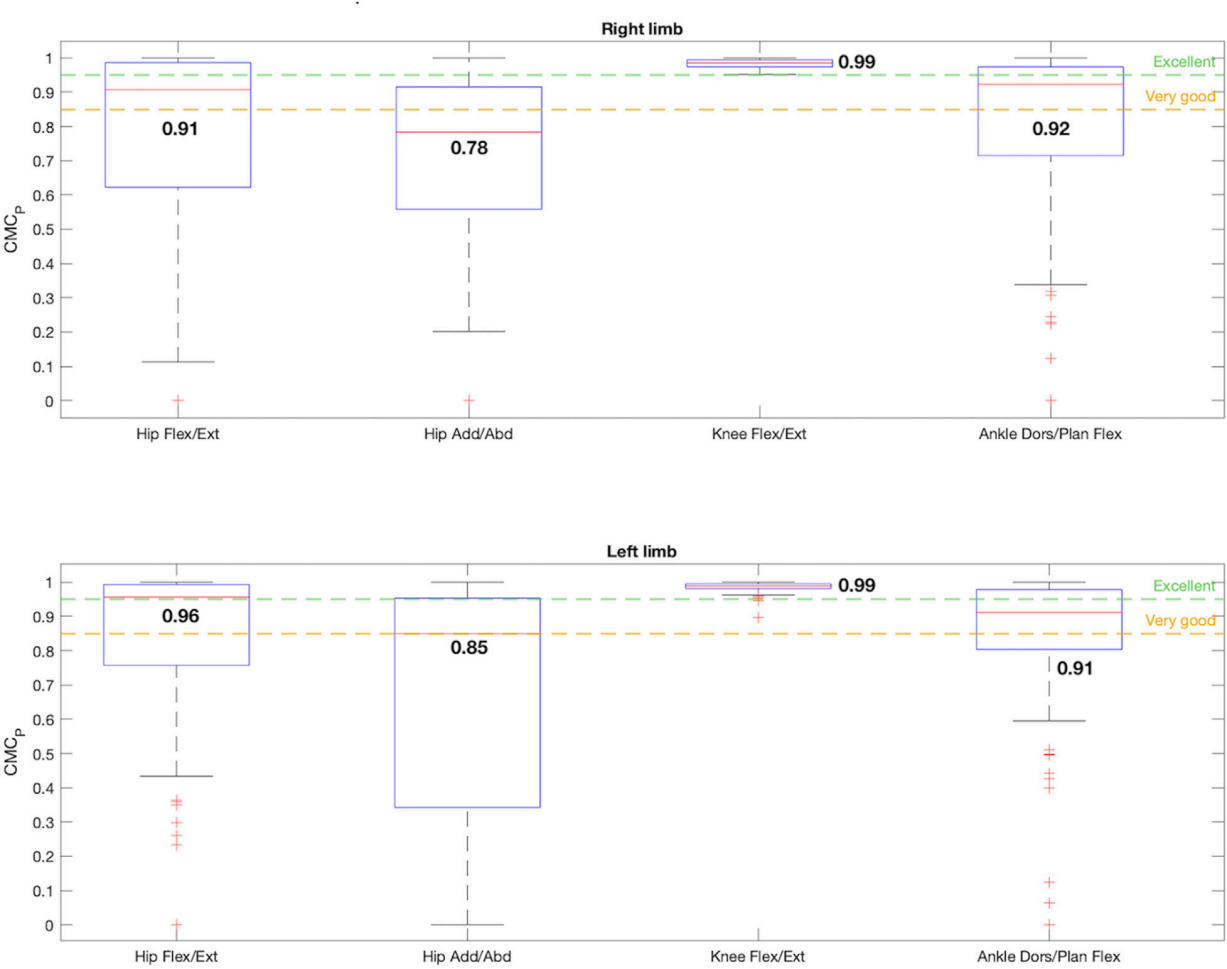
Box-and-whisker plot for CMC_P_ regarding the comparison between the 4 joint angles that satisfy condition 1: hip flexion/extension, hip adduction/abduction, knee flexion/extension and ankle dorsi/plantar flexion. The median value for each joint angle is reported.

In terms of the CMC_P_ calculation (Figure 6), whose formula shown in Equation 4 included the whole effect of the offset, r, and ∆ROM, the knee flexion/extension movement was the only one that reached an excellent median value of 0.99 for both limbs with hardly any data dispersion, satisfying condition 2. For the hip in the sagittal plane, the left one also fulfilled condition 2, displaying an excellent median CMC_P_ value of 0.96. The right hip also presented a very good CMC_P_ of 0.91 between pairs of [I(t), V(t)]. For the frontal plane, the right hip had a good CMC_P_ value of 0.78, whereas the left one reached a very good coefficient value of 0.85. Lastly, both ankles presented median CMC_P_ values around 0.9, showing a very good similarity.

**FIGURE 7:**
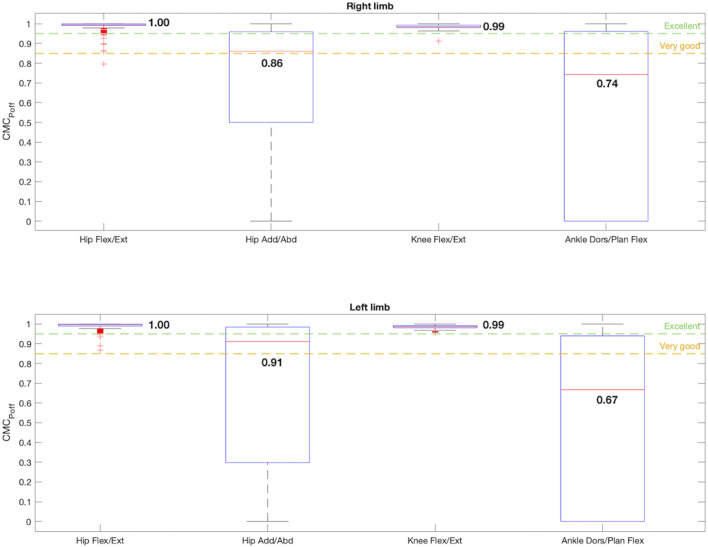
Box-and-whisker plot for CMC_Poff_ regarding the comparison between the 4 joint angles that satisfy condition 1: hip flexion/extension, hip adduction/abduction, knee flexion/extension and ankle dorsi/plantar flexion. The median value for each joint angle is reported.

The effects of correcting the offset are shown in Figure 7. Once the offsets between corresponding waveforms were removed, the CMC_Poff_ values improved for every joint angle, except for the ankles dorsi/plantar flexion (right: CMC_Poff_ = 0.74, left: CMC_Poff_ = 0.64), whose data dispersion also increased considerably. For both limbs in the sagittal plane the hips and knees flexion/extension described an excellent similarity of 1 and 0.99, respectively. The dispersion of the data enhanced significantly after removing the offset for the hips in the sagittal plane. The hips CMC_Poff_ also increased in the frontal plane, becoming 0.86 for the right limb and 0.91 for the left one, both in the very good similarity range. Therefore, when the offset was corrected, condition 2 was fulfilled by hip flexion/extension and adduction/abduction, and by knee flexion/extension for both limbs.

**FIGURE 8:**
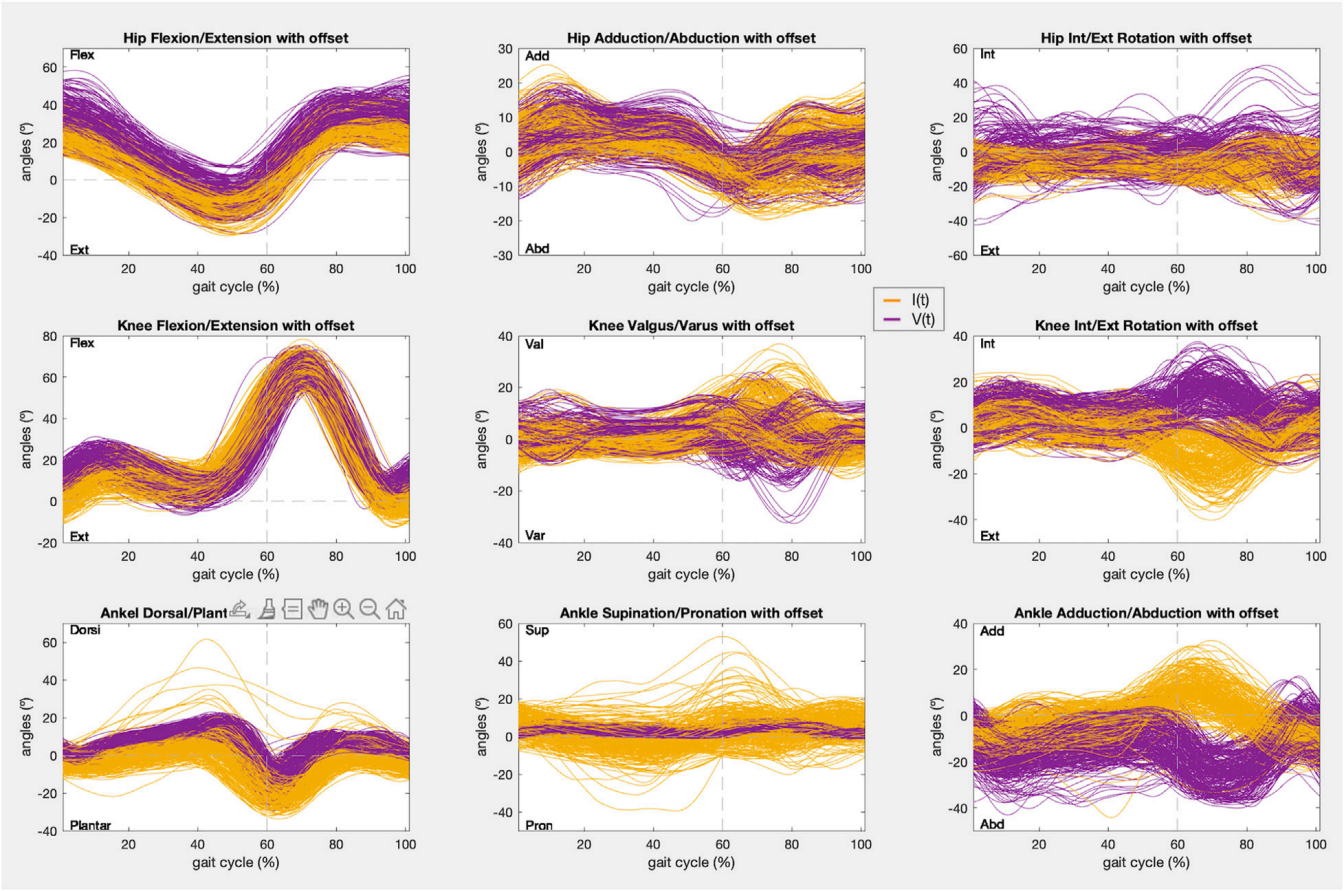
Right limb kinematic pattern of the gait cycles registered with IMUs (yellow) and the photogrammetry system (purple) in the 9 joint angles.

**FIGURE 9:**
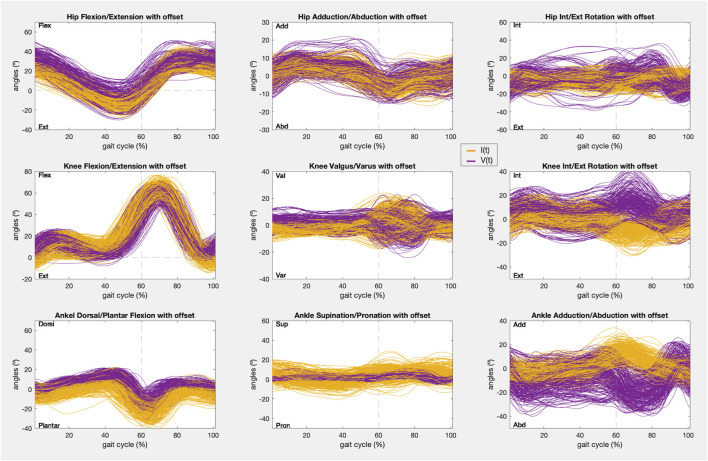
Left limb kinematic pattern of the gait cycles registered with IMUs (yellow) and the photogrammetry system (purple) in the 9 joint angles.

**FIGURE 10:**
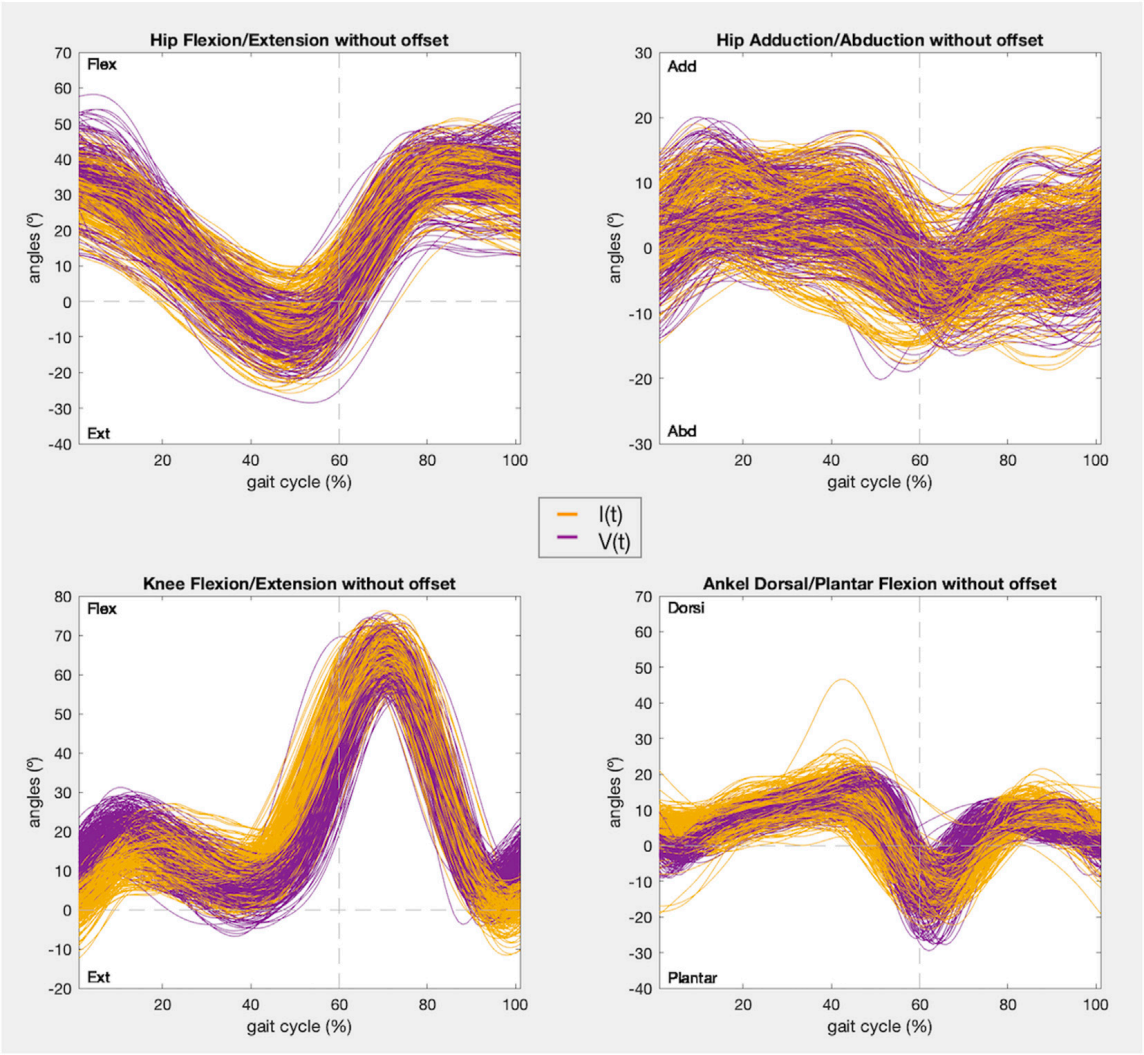
Right limb kinematic pattern after offset zeroing of the gait cycles registered with IMUs (yellow) and the photogrammetry system (purple) in the 4 joint angles that satisfy condition 1.

**FIGURE 11:**
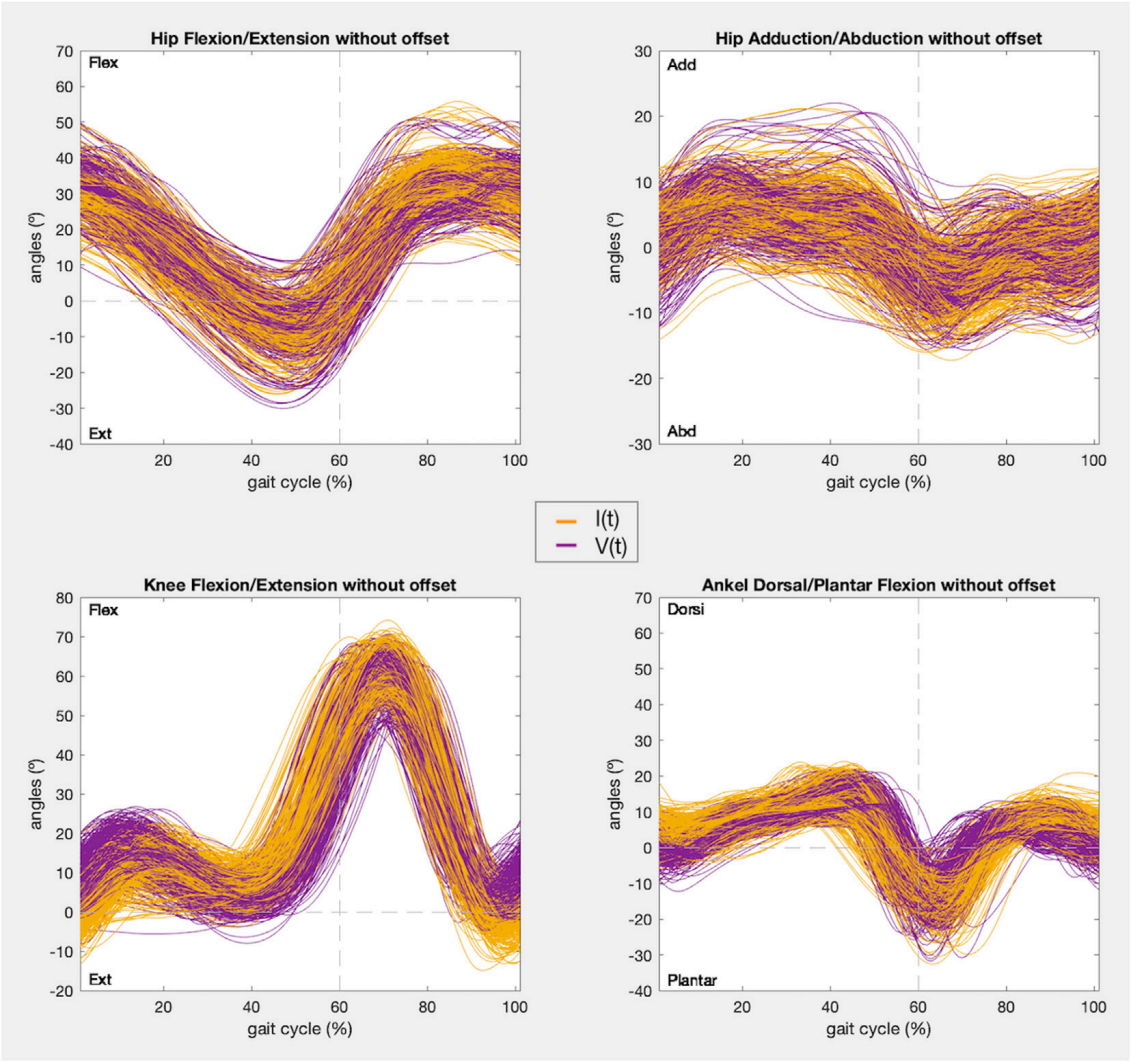
Left limb kinematic pattern after offset zeroing of the gait cycles registered with IMUs (yellow) and the photogrammetry system (purple) in the 4 joint angles that satisfy condition 1.

In addition, the kinematic pattern of each pair of gait cycles [I(t), V(t)] for the 9 joint angle is shown in Figures 8, 9, containing kinematic data of the right and left limbs, respectively. Lastly, the kinematic pattern with the offset arrangement is displayed only for those joint angles that have been analyzed in condition 2. Figure 10 shows the offset correction for the right limb joints and Figure 11 for the left ones.

## 4 Discussion

The kinematic pattern of the hip flexion/extension and adduction/abduction, the knee flexion/extension and the ankle dorsi/plantar flexion were equivalent when captured by each mocap system synchronously. However, when the offset was corrected, only the hip flexion/extension, the hip adduction/abduction and the knee flexion/extension satisfied the conditions to be considered similar with high confidence.

Although the CMC_P_ included the effect of every estimated parameter, it was considered necessary to study individually the variability, trend, and dispersion of each of them to properly evaluate the accuracy of the synchronous pairwise measurements [I(t), V(t)]. Likewise, it was decided to study each parameter individually to identify the root cause of the differences in the measurements registered by each mocap system, since some of them could be controlled by adjusting the IMUs instrumentation model or the registration protocol, thus improving the precision of the movement recorded with the IMUs.

Initially, given the limitations observed in CMC in previous papers and in this work, it was decided to perform an initial analysis focused uniquely on the assessment of the correlation-centered waveform similarity (Growney et al., 1997; Steinwender et al., 2000; Røislien et al., 2012). If the waveforms of each pair of gait cycles [I(t), V(t)] did not maintain a linear dependence relationship, that is, they did not change in the same way, it was assumed that the kinematic pattern was not similar, and the in-depth analysis was not performed.

As stated in the results, the only joint angles that showed a sufficiently strong correlation (r > 0.75) to meet condition 1 were the movements of all joints (hips, knees, and ankles) in the sagittal plane and the hips in the frontal plane (Figure 2). In addition, these joint boxes showed less data dispersion and thus less variability in the recordings. Furthermore, this result is confirmed by the graphs in Figures 8, 9, which show the kinematic pattern of all the pairs of gait cycles registered with the IMUs and the photogrammetry system for all joint angles. It can be observed that all the signals in the sagittal plane and the hips in the frontal plane follow the same waveform behavior with both systems. Some outliers are also observed in the dorsi/plantar flexion of the right ankle, showing a registration error by the IMUs. The hypothesis of the origin of this error is the distortions of the magnetometers and the drift accumulated during the captures.

Besides, it can also be seen that the IMUs and photogrammetry signals of the remaining joint angles did not have the same waveform, confirmed by their poor correlation (r < 0.65). In other studies it has been demonstrated that the kinematics recorded in the sagittal plane in adult gait are the most repeatable within the same day and between days, while the repeatability of gait kinematic pattern in the other planes is much more variable for the same subject even within the same day, mainly in the transverse plane for every joint and knee valgus/varus (Kadaba et al., 1989; Growney et al., 1997; Besier et al., 2003; Mcginley et al., 2009). In this study, this can be appreciated especially in hip rotation and knee valgus/varus movements (Figures 8, 9), where the average kinematic pattern recorded with photogrammetry does not describe a common trend, showing more data variability, and does not correspond to that defined in the theory (van der Linden, 2011; Duarte et al., 2018; Francisco and Tejada, 2020). Additionally, there is evidence that, despite their accuracy, photogrammetry systems are less reliable in the transverse plane and knee varus/valgus during gait analysis (Fonseca et al., 2023). In fact, most studies report the highest errors in those planes (Growney et al., 1997; Kim and Nussbaum, 2013; Kok, Hol, and Schön, 2014). So, adjusting the accuracy of other less robust devices in these planes based on the similarity assessment could not be as reliable.

The second part of the analysis focused on the four planes that satisfied condition 1. The position of each IMU sensor was defined so that it would record the ROM as completely as possible. Looking at Figure 3, the ∆ROM recorded for hips flexion/extension, hips adduction/abduction and ankles dorsi/plantar flexion barely reached 2°. These values are closed to those obtained in similar IMUs and photogrammetry systems validation studies (Picerno, Cereatti, and Cappozzo, 2008; Ferrari et al., 2010b) and, in addition, fall within the ranges considered despicable according to the minimal clinically important differences (MCID) estimated for unaffected limbs (Guzik et al., 2021). The knee flexion/extension difference values reached 6.8° in the left limb, higher than those reported in the mentioned validation studies. However, this is still a negligible value with respect to the MCID estimated in clinical practice for unaffected limbs in other articles, which almost reaches 7° (Guzik et al., 2020). It should also be noted that previous assessment studies include samples of one to four subjects, whereas in this work the sample was increased to 32 users, therefore, more variability was captured in the dataset.

Data from the articles reporting errors reveal that most of the studies and gait variables present errors between 2° and 5° for every joint angle, although few of them reach the sample size presented here. The lower RMSE obtained in this validation, in Figure 5, was the one corresponding to the hips adduction/abduction, whereas in the sagittal plane the knees reached 7° and the hips and the ankles reached 10°. However, there are also studies reporting errors between 5° and 10° in the sagittal plane during clinical assessment in the lower limb (McDowell et al., 2000; Fosang et al., 2003). In this study, therefore, these errors can be considered negligible.

As explained in the methodology, the mode of computing CMC_P_ is significantly influenced by joint ROM. Previous studies note that joints with a large ROM tend to record high CMC_P_ and, conversely, joints with a low ROM tend to show lower reliability (Growney et al., 1997; Steinwender et al., 2000; Mcginley et al., 2009; Røislien et al., 2012). This limitation was also shown in this validation (Figure 6), because the lowest CMC_P_ values were reported for the joint angles with smaller ROMs evaluated which were hip adduction/abduction (CMC_P_ = 0.8) and the ankle dorsi/plantar flexion (CMC_P_ = 0.9), even though the measured error and waveform correlation values obtained were good. However, in this study the reliability value increased in the frontal plane because most studies report minimum reliability indices of 0.7. This could be thanks to the standardized instrumentation proposed for the IMUs, defined following the anthropometry measurements to enhance reliability. For the hip and knee in the sagittal plane, the best repeatability and reliability values were obtained (CMC_P_ > 0.9), as reported in the other articles, confirming the feasibility of recording precise kinematics of this plane with IMUs (Mcginley et al., 2009; Ferrari et al., 2010b; Robert-Lachaine et al., 2017).

On the other hand, the CMC_Poff_ was computed to assess how the offset affects the calculation of the repeatability (Figure 7). Once removed, the CMC_Poff_ of the hips in the sagittal plane became excellent, this movement was the one with more offset registered (right: offset = −9.06°; left: offset = −7.98°), and in the frontal plane it increased to 0.9, demonstrating that reliability was directly influenced by the offset. The dispersion of the data, and therefore its variability, improved significantly. Meanwhile, the knees were not disturbed by the offset, whose value is 1°, since they maintained an excellent reliability of 0.99. On the contrary, the CMC_Poff_ of the ankles decreased once the offset is removed. As the CMC_P_ includes the effect of the r, the offset and ∆ROM, if the CMC_Poff_ did not improve when correcting the offset, the root of the problem is another of these parameters. However, as previously stated, the values of ∆ROM, RMSE and r were good. In addition, it was found that most of the values obtained for the [I(t), V(t)] couples were complex, thus, the hypothesis is that the CMC_Poff_ estimation was limited in this movement because of the small ROM of this joint angle, resulting in the formula breaking down (Røislien et al., 2012). As there is no consensus for interpreting these complex values, the acceptance of ankle flexion/extension as reliable was centered on all the other parameters studied. For the CMC_P_ with offset included, this plane showed a value of 0.92 and 0.91 for right and left extremities respectively, showing a very good reliability range, despite not fulfilling condition 2.

According to the IMUs instrumentation protocol, it is known that the offset is caused due to two main reasons: the difference in degrees introduced due to anatomical morphology and the difference in degrees introduced during calibration. In both cases these introduced degrees remained constant during all the captures for the same subject. Likewise, both hips and ankles showed a constant offset for all the subjects, as can be seen in Figures 4, 8, 9, and in the kinematic patterns whose offsets were corrected, shown in Figures 10, 11. Considering that it could be arranged in post-processing to achieve a satisfactory reliability for gait recordings with IMUs, it was agreed that values obtained with CMC_Poff_ satisfy the requirements established in the methodology.

For experiments in which the IMUs mocap system is used to register healthy adults kinematic gait data, it is proposed to perform the offset correction in post-processing by subtracting to the whole signal the median offset value obtained in this study for each joint angle, shown in Figure 4, or by measuring with a goniometer the offsets of each joint angle intrinsic to anatomical morphology that could be observed before starting the recording.

## 5 Conclusion

The kinematic pattern of the hip and the knee in the sagittal plane and the hip in the frontal plane satisfies condition 2 showing an excellent and a very good similarity, respectively, between the waveforms captured with the IMUs and those captured with the photogrammetry system synchronously. Therefore, it is concluded that the movements registered by the IMUs in those planes are completely reliable with offset included. However, the reliability increases if the offset correction is performed, especially for the hip adduction/abduction movement. On the other hand, the ankle dorsi/plantar flexion shows a very good range of similarity between the IMUs and photogrammetry records. Although it does not fulfill condition 2, it can be used to obtain measurements of this joint angle with very good reliability.

It is also concluded that standardizing the instrumentation of the IMUs to the anthropometric measurements of each subject favors the complete recording of the ROM of each joint. However, this is not enough to adjust the offset control, since it also depends on a good calibration position in which the joint angles are aligned at 0°.

## Data Availability

The raw data registered for this study can be found in Zenodo, an open research data repository, at this link https://doi.org/10.5281/zenodo.11242602.
